# Case Report: Possible spatially-distinct *de novo* invasive cervical carcinoma in the setting of isolated persistent HPV16 after ALA-PDT-mediated histological clearance of CIN3

**DOI:** 10.3389/fmed.2026.1954126

**Published:** 2026-09-08

**Authors:** Huihui Cai, Yanci Che, Yutong Liu, Jing Wang, Tingting Ma, Rui Yao

**Affiliations:** 1 Department of Gynecology, Laoshan Branch, Affiliated Hospital of Qingdao University, Qingdao, China; 2 Medical Record Management Center, Affiliated Hospital of Qingdao University, Qingdao, China; 3 College of Resource Environment and Tourism, Hubei University of Arts and Science, Xiangyang, China

**Keywords:** ALA-PDT, case report, CIN3, *de novo* cervical carcinoma, fertility preservation, loss to follow-up, persistent HPV16, stratified surveillance

## Abstract

**Background:**

Cervical intraepithelial neoplasia grade 3 (CIN3) is a high-grade precancer, and 5-aminolaevulinic acid-mediated photodynamic therapy (ALA-PDT) offers a fertility-preserving alternative for young patients. However, complete lesion regression does not eliminate the latent oncogenic risk of persistent HPV16, and real-world evidence of invasive carcinoma arising after long-term loss to follow-up (LTFU) post-PDT remains scarce. This case report aims to share this clinical observation and provide real-world evidence supporting risk-stratified post-treatment surveillance for patients with isolated persistent HPV16 after histological CIN3 remission.

**Case presentation:**

A 32-year-old parous woman with TZ2 CIN3 at the 9/12 o'clock cervical positions achieved histological remission after two cycles of standardized ALA-PDT, yet retained isolated HPV16 infection. Despite repeated clinical reminders, she discontinued all surveillance for 20 consecutive months. Upon voluntary re-presentation, newly developed invasive squamous cell carcinoma was confirmed at the 6 o'clock position—a cervical quadrant spatially distinct from the original CIN3 lesions. This finding raises the possibility of *de novo* malignant transformation from persistent HPV16; however, pre-existing occult multifocal disease cannot be excluded in the absence of viral clonality or HPV integration-site molecular evidence. Multimodal staging indicated theoretical eligibility for fertility-sparing radical trachelectomy.

**Conclusions:**

Isolated persistent HPV16 retains long-term malignant potential despite resolved precancerous lesions, and can drive spatially separate invasive carcinoma. Because this inference is based on a single observational case without supporting molecular data, risk-stratified intensified surveillance should be further validated in larger cohorts. Standardized follow-up adherence interventions are required to prevent irreversible fertility loss and advanced disease secondary to discontinuous monitoring.

## 1 Introduction

High-risk human papillomavirus 16 (HPV16) is the dominant oncogenic genotype driving most high-grade squamous intraepithelial lesions (HSIL) and invasive cervical cancer (1, 2). As summarized in a recent narrative review exploring the complex interplay between cervical intraepithelial neoplasia and human papillomavirus infection, persistent high-risk HPV infection constitutes the necessary biological prerequisite for the vast majority of cervical precancerous and malignant events (3). For cervical intraepithelial neoplasia grade 3 (CIN3), cold-knife conization and loop electrosurgical excision procedure (LEEP) remain the gold-standard interventions, while 5-aminolaevulinic acid-mediated photodynamic therapy (ALA-PDT) serves as an off-guideline organ-preserving alternative for patients with urgent fertility demands (4–7). Retrospective and prospective cohorts document 80%–89% short-term complete remission (CR) rates of CIN3 after standardized ALA-PDT, yet 10%–28% treated individuals retain persistent high-risk HPV, predominantly HPV16 (8–11); notably, even in low-grade squamous intraepithelial lesions (LSIL) cohorts, ALA-PDT achieves 80%–94% short-term HPV clearance yet a subset of patients retains persistent infection requiring long-term surveillance (12). Type 2 transformation zone (TZ2) lesions carry significantly higher post-treatment HPV persistence rates compared with TZ1/TZ3 cases (9, 13).

Published molecular-sequencing studies have demonstrated that integrated HPV16 fragments can persist within morphologically normal cervical epithelium for years after visible dysplasia clearance, disrupting tumor-suppressor genes and establishing a latent carcinogenic microenvironment in other patient cohorts (14–17). No comparable longitudinal molecular data were available for the present case to verify such viral integration events in this individual patient. Loss of HPV L1 protein expression accompanying viral genome integration limits the diagnostic performance of conventional cytology to identify subclinical latent infection, creating a monitoring blind spot for patients with normalized histology after lesion ablation (18, 19). Global screening programs record high long-term loss to follow-up (LTFU) rates (40%–69%) among HR-HPV-positive precancer patients, largely attributable to patient misconceptions that negative short-term histology equates to permanent cure (20–22). Publications describing *de novo* invasive carcinoma arising solely from long-standing isolated HPV16 after full CIN3 histological remission remain extremely limited (23–25), though Frontiers in Medicine has published cervical cancer case reports underscoring the diverse clinical presentations of HPV-related malignancies (26).

This case report presents a unique clinical observation: a fertility-preserving patient with no residual CIN3 detected on post-PDT biopsy for TZ2 CIN3 who developed spatially distinct invasive cervical carcinoma following 20 consecutive months of unmonitored persistent HPV16 infection, providing critical real-world evidence for risk-stratified post-PDT surveillance.

## 2 Case presentation

**Figure 1:**
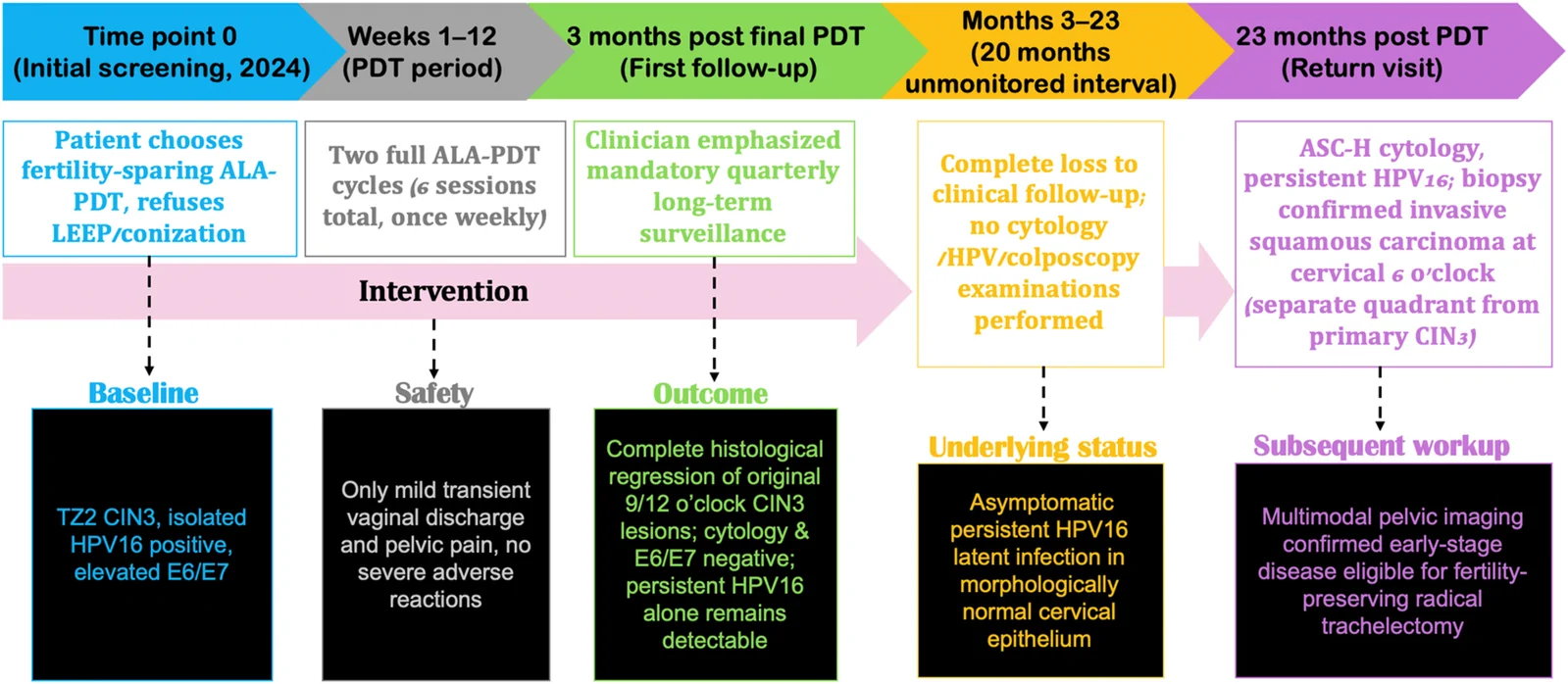
Chronological clinical timeline of the patient's disease course. Five core stages: (1) baseline TZ2 CIN3 diagnosis with isolated HPV16 at 9/12 o'clock; (2) two complete ALA-PDT cycles; (3) 3-month post-treatment verification showing no residual CIN3 on biopsy but persistent HPV16; (4) 20-month loss to follow-up; (5) *de novo* invasive carcinoma at the 6 o'clock position. Key findings at each time node illustrate the clinical course; true *de novo* carcinogenesis cannot be confirmed and pre-existing occult disease cannot be excluded.

The full clinical timeline is presented in Figure 1. Given the patient's strong desire for fertility-preserving non-excisional management, ALA-PDT was selected as an off-guideline therapeutic alternative. Pre-treatment counselling comprehensively discussed standard therapeutic alternatives including LEEP and cold-knife conization. We also explicitly communicated the substantial oncological risks if the patient declined all active treatment and opted for observation alone; surveillance-only observation is not regarded as a standard therapeutic option for non-pregnant patients with CIN3. The patient was explicitly informed that ALA-PDT is not formally recommended as standard first-line therapy for CIN3 per current Chinese Society for Colposcopy and Cervical Pathology (CSCCP) guidelines, together with associated risks including persistent or recurrent high-grade cervical lesions and potential risk of disease progression. Written informed consent for this off-guideline photodynamic therapy was obtained prior to initiation of treatment.

### 2.1 Baseline presentation and ALA-PDT treatment

A 32-year-old parous female (G1P1) received opportunistic cervical screening in 2024. Initial testing returned HSIL cytology, positive HPV16, and elevated HPV E6/E7 oncoprotein levels. Colposcopy at an external tertiary hospital identified TZ2 anatomy, with targeted biopsy at the 9 and 12 o'clock cervical positions confirming CIN3; pathological slide consultation at our center validated this diagnosis (8, 11). Per 2023 CSCCP guidelines, TZ2 satisfies PDT technical eligibility, yet CIN3 is not formally indicated for ALA-PDT, with LEEP and conization as standard procedures (5, 6). In this case, standard excisional treatment options, expected therapeutic benefits, recurrence risks, and oncological hazards associated with this off-guideline ablative approach were comprehensively communicated during pre-treatment counselling. The patient voluntarily chose ALA-PDT and declined excisional surgery, with written informed consent documented. She completed all six PDT sessions [two full cycles of topical 20% 5-ALA followed by 635 nm diode laser irradiation per institutional standards (8, 23, 27)] without severe adverse events; mild vaginal discharge and transient pelvic cramping resolved spontaneously within 72 h.

Supplemental technical parameters for reproducibility: 20% 5-ALA dispersant solution (Shanghai Fudan-Zhangjiang Bio-Pharmaceutical Co., Ltd., China) was freshly prepared prior to each session. The cervical canal was packed with ALA-soaked tampon, and cervical lesion surfaces were fully covered with ALA-soaked cotton swabs for 4 h of dark incubation before light irradiation. Irradiation was performed using an LD600 diode laser system (Wuhan Yage Optics and Electronic Technique Co., Ltd., Wuhan, China) at a wavelength of 635 nm, with a power density of 80 mW/cm^2^; the optical fiber was placed 2 cm away from the cervical epithelium, delivering a total irradiation duration of 25 min per session. The treatment field fully covered the entire cervical transformation zone. Individual PDT sessions were administered at a 7-day interval; the patient completed two full treatment cycles (six total PDT sessions) according to our institutional protocol, consistent with our previously published retrospective cohort study of ALA-PDT for CIN3 (8). The calculated irradiation fluence was 120 J/cm^2^. According to the routine clinical setting for cervical PDT using the LD600 diode laser system, the spot diameter was set to 2 cm (standard-mode factory specification; alternative large-spot mode = 4 cm).

All cervical-screening, colposcopy, and ALA-PDT operational procedures followed national expert operation specifications for cervical photodynamic therapy. Complete histological remission was determined at the 3-month post-treatment follow-up visit by combined colposcopic assessment and colposcopy-directed punch biopsies targeting the prior lesion area and all visually abnormal cervical sites. Complete absence of high-grade squamous intraepithelial lesion on histopathology was defined as complete histological remission. Of note, ALA-PDT is not formally recommended as standard first-line therapy for CIN3 per current CSCCP guidelines; excisional modalities including LEEP or cold-knife conization remain the guideline-endorsed standard of care (5, 6). Nevertheless, accumulating real-world cohort data have demonstrated satisfactory short-term histological remission rates for carefully selected CIN3 patients receiving ALA-PDT who desire fertility preservation (8).

### 2.2 Three-month post-PDT surveillance

Three months post-PDT, routine colposcopy with targeted biopsy verified no residual CIN3 detected on post-treatment colposcopy-directed biopsies. Liquid-based cytology showed no atypical squamous cells, and HPV E6/E7 testing yielded negative results. HPV E6/E7 mRNA testing was performed using the High-risk Human Papillomavirus E6/E7-region mRNA Detection Kit (PCR-fluorescent probe assay, HybriBio, Chaozhou, China; NMPA registration No. 20233401459), following the manufacturer's instructions. Notably, a negative E6/E7 result cannot fully exclude oncogenic activity within unsampled cervical epithelium due to sampling limitations and variable viral transcriptional status. However, isolated HPV16 persisted without co-existing other high-risk subtypes. Clinical providers delivered standardized patient education on mandatory quarterly to semi-annual monitoring and the sustained latent carcinogenic capacity of unresolved HPV16 (1, 2). Despite consistent telephone and outpatient reminders, the patient declined all subsequent surveillance appointments for 20 consecutive months (20, 22).

### 2.3 Malignant progression and fertility-sparing assessment

Upon voluntary re-presentation, repeat cytology demonstrated ASC-H with unchanged isolated HPV16 positivity. Diagnostic colposcopy with targeted biopsy at the cervical 6 o'clock lower lip confirmed invasive squamous cell carcinoma—localized to a cervical quadrant entirely distinct from the original 9/12 o'clock CIN3 lesions. At baseline, the entire TZ2 transformation zone was adequately visualized, and baseline endocervical curettage yielded negative results for dysplasia. The 6-o'clock site showed no colposcopic abnormality at baseline; nevertheless, tiny occult microscopic lesions cannot be ruled out by visual assessment alone. Baseline colposcopic images are archived and available for retrospective review. Even with these reassuring baseline findings, pre-existing micro-scale occult multifocal disease cannot be completely excluded. This spatial separation raises the possibility of PDT-mediated clearance of the primary precancerous lesion; nevertheless, pre-existing occult multifocal disease cannot be fully excluded given the absence of viral clonality or HPV integration-site molecular evidence.

Comprehensive multimodal preoperative staging was completed: all serum tumor markers within normal ranges; hysteroscopy visualized a localized cervical mass without endocervical extension; transvaginal ultrasound detected no abnormal neovascular signals; and multiparametric pelvic MRI demonstrated heterogeneous enhancement within the cervical region, without enlarged pelvic lymph nodes or parametrial dissemination. The patient declined radical trachelectomy performed at our institution and sought fertility-sparing surgical care at Peking Union Medical College Hospital. She underwent conization with a resection depth of 2 cm, performed for both diagnostic and potential fertility-sparing therapeutic purposes. Post-conization surgical-pathological findings confirmed invasive squamous cell carcinoma: tumor size 2 cm; stromal invasion depth 7 mm; horizontal extent: 2 cm; moderately differentiated histology; p16 diffuse positive; Ki-67 proliferation index 80%; lymphovascular-space invasion positive; FIGO 2018 stage IB2. The surgical resection margin was negative, with no dysplasia or invasive carcinoma identified at the margin. Multidisciplinary discussion at the external institution recommended total hysterectomy given the presence of lymphovascular-space invasion despite negative surgical margins; this definitive surgical procedure is scheduled at the end of the current month and has not yet been performed at the time of manuscript preparation. Lifelong continuous oncological surveillance will be pursued following definitive surgery. Representative raw colposcopic and histopathological images could not be included in this report because the patient declined consent for release of these clinical photographs.

### 2.4 Diagnostic assessment

Formal institutional review board ethical clearance (QYFY WZLL 50314) was granted prior to data collection. Full written informed consent was obtained for publication of de-identified clinical, cytological, histopathological, hysteroscopic and MRI data. All cervical-screening procedures, colposcopy techniques, and technical execution of ALA-PDT followed 2022-2026 CSCCP operational recommendations and national cervical PDT expert operation specifications (4, 5, 7, 28, 29). Note that ALA-PDT is not a guideline-endorsed indication for CIN3 in this setting. Baseline screening included TCT, full HR-HPV genotyping, and HPV E6/E7 oncoprotein detection (30, 31).

## 3 Discussion

This case describes a possible spatially-distinct subsequent invasive carcinoma occurring under conditions of isolated persistent HPV16 after histological clearance of index CIN3 lesions following ALA-PDT. We cannot rule out an alternative explanation: pre-existing, occult, unsampled multifocal cervical disease may have been present at baseline and escaped detection by targeted biopsies limited to the 9- and 12-o'clock positions. Spatial separation of lesions alone is insufficient to confirm true *de novo* malignant transformation in the absence of viral clonality or HPV integration-site molecular evidence.

ALA-PDT represents an ablative rather than excisional therapeutic modality. Negative post-treatment targeted biopsies only reflect sampled tissue sites and cannot rule out occult disease across the whole cervical epithelium.

The patient experienced a 20-month interval without clinical surveillance while HPV16 infection persisted. This unmonitored period permitted disease progression to escape clinical detection. Nevertheless, we cannot determine the exact timeline of malignant transformation purely based on this single observational case; loss to follow-up delayed diagnosis rather than directly causing carcinogenesis.

While existing literature supports biologically plausible mechanistic frameworks linking persistent HPV-16 infection, viral integration and clonal evolution to cervical carcinogenesis, none of these molecular events were directly demonstrated in the present patient owing to the unavailability of patient-specific viral sequencing and clonality assays.

Complete lesion remission does not eliminate latent oncogenic risk. Published nanopore-sequencing studies from other cohorts indicate that integrated HPV16 genomes may retain oncogenic activity within morphologically normal cervical epithelium for years after ablation of visible dysplastic lesions (14, 15, 17). However, we lack patient-specific viral-sequencing data to confirm this molecular mechanism occurred in our case. As described in published work, HPV16 E6/E7 oncoproteins can continuously inhibit p53 and retinoblastoma signaling even in the absence of histologic abnormalities (18, 19). Published PDT adverse outcomes predominantly document residual primary precancerous tissue rather than secondary malignancy at separate cervical sites (24, 32, 33). This case raises the possibility of spatially-distinct subsequent carcinoma associated with persistent HPV16, though definitive *de novo* malignant transformation cannot be proven given the absence of patient-specific molecular evidence.

Second, LTFU poses severe clinical hazards for reproductive-age women. Global screening data record 41%–69% LTFU among HR-HPV-positive precancer patients (20, 22). Current guidelines implement uniform follow-up intervals for all post-PDT patients, lacking tailored intensified monitoring specifically for isolated persistent HPV16 carriers (5, 29); systematic reviews of HPV screening modalities within primary care further highlight the critical need for optimizing follow-up strategies to improve detection of high-grade lesions among infected women (34).

Third, risk-stratified surveillance is urgently needed. We put forward the following as a hypothetical risk-stratified surveillance strategy requiring validation in larger patient cohorts, rather than formal clinical recommendations derived solely from this single-case report (1): Intensified monitoring for persistent HPV16 carriers: quarterly combined TCT-HPV-colposcopy for the initial three post-treatment years, transitioning to semi-annual monitoring from year four to year ten, distinct from standard intervals for HPV-negative patients after treatment (35, 36). We acknowledge that such high-frequency surveillance imposes substantial clinical-visit burden on patients, and real-world adherence to quarterly colposcopy-based surveillance is often suboptimal. Individualized balancing between surveillance intensity, patient adherence, and clinical resource availability will be necessary in routine practice. Definitive surveillance intervals for this specific population await prospective multicenter data, while routine patient management should follow current evidence-based clinical guidelines (2). Multimodal adherence promotion: automated digital reminders, dedicated nurse coordinators for high-risk individuals, and expanded low-barrier community screening access (20, 22); (3) Targeted patient education: standardized counseling correcting the misconception that negative biopsy equals permanent cure, explicitly communicating multi-year latent cancer risk (20, 25). Clinicians should base routine patient management on existing evidence-based guidelines; further prospective studies are required to confirm whether patients with isolated persistent HPV16 after ALA-PDT truly benefit from intensified surveillance. Adjuvant HPV vaccination has been validated to improve viral clearance and reduce recurrence after cervical dysplasia treatment, providing a potential combinative intervention strategy following ALA-PDT (37); moreover, DNA methylation biomarkers have been identified as predictive indicators for ALA-PDT treatment outcomes in cervical intraepithelial lesions (38), which may serve as molecular tools for risk stratification in post-PDT surveillance.

### 3.1 Limitations

This single-case observation lacks population-level quantitative risk estimates; large multicenter cohorts are required to calculate absolute long-term malignant incidence (23, 24). Several key limitations deserve emphasis. First, we have no patient-specific longitudinal molecular data, including HPV integration-site sequencing and viral clonality testing. Therefore, the anatomical spatial separation between the index CIN3 lesion and the subsequent invasive carcinoma cannot serve as definitive proof of true *de novo* malignant transformation, and the alternative hypothesis of unrecognized baseline occult multifocal cervical disease remains clinically plausible. Second, PDT is an ablative therapy without resected surgical specimens; negative post-treatment targeted biopsies only reflect sampled sites and cannot completely exclude occult disease elsewhere within the cervical epithelium. Third, patient follow-up compliance varies across healthcare-resource levels, limiting broad generalizability (22). Fourth, the surveillance strategy proposed in this manuscript is hypothesis-driven from clinical observation and requires external validation and should not be adopted as formal clinical recommendations based solely on this report. Future multicenter longitudinal studies are warranted to develop molecular risk biomarkers based on HPV-induced microRNA signatures for risk stratification (39). Additionally, definitive total hysterectomy has been planned but not completed at the time of manuscript submission. Therefore, complete postoperative pathological evaluation from the hysterectomy specimen, including further assessment for residual disease and lymphovascular-space status, remains unavailable.

### 3.2 Patient perspective

The patient expressed deep regret regarding the discontinuation of follow-up appointments, acknowledging that she had misinterpreted the 3-month negative pathology results as definitive cure. She stated: “If I had understood that persistent HPV16 could still cause cancer even after the lesion disappeared, I would not have skipped my check-ups.” She consented to publication of this case specifically to alert other young women that normal post-treatment histology on biopsy does not guarantee permanent safety when HPV16 remains detectable, even though we cannot conclusively distinguish true *de novo* transformation from progression of pre-existing occult microscopic disease.

## 4 Conclusions

ALA-PDT delivers reliable histological remission of TZ2 CIN3 on sampled biopsy sites for reproductive-age women. Nevertheless, isolated persistent HPV16 retains severely under-recognized long-term oncogenic potential. This observational case illustrates that a prolonged period of unmonitored chronic HPV16 infection may be associated with subsequent invasive cervical carcinoma at a spatially separate cervical site, after no residual CIN3 was detected by post-treatment biopsy. Owing to the absence of supporting molecular evidence, we cannot draw definitive causal conclusions or formally confirm true *de novo* carcinogenesis. Intensified risk-stratified monitoring and standardized adherence interventions are urgently required. Large prospective multicenter research is warranted to refine personalized long-term surveillance guidelines.

## Data Availability

The raw data supporting the conclusions of this article will be made available by the authors, without undue reservation.

## References

[1] Lee J Kim DJ Lee HJ. Assessment of malignant potential for HPV types 16, 52, and 58 in the uterine cervix within a Korean cohort. Sci Rep. (2024) 14:14619. 10.1038/s41598-024-65056-7 38918416 PMC11199604

[2] Dushkin A Grishacheva T Afanasiev S Dushkina I Karaulov A Biryukova E. Photodynamic therapy efficacy of the human papillomavirus-related cervical lesions. J Clin Med. (2026) 15:1596. 10.3390/jcm15041596 41753289 PMC12941384

[3] Karimi P Hosseini SMR Mousavian Hiagh ZS Aboulhassanzadeh S Asghari N Aghazadeh H. Exploring the intricacies of cervical intraepithelial neoplasia and its connection with HPV: a narrative review. Int J Prev Med. (2024) 53:17310. 10.18502/ijph.v53i12.17310 PMC11693788 39759206

[4] Chinese Society for Colposcopy and Cervical Pathology (CSCCP). Chinese expert consensus on management of cervical high-grade squamous intraepithelial lesions. Chin J Clin Obstet Gynecol. (2022) 23:220–4. 10.13390/j.issn.1672-1861.2022.02.038

[5] CSCCP. Guidelines for cervical cancer screening in China (part I). Chin J Clin Obstet Gynecol. (2023) 24:437–42. 10.13390/j.issn.1672-1861.2023.04.029

[6] Committee of Gynecological Oncology of Chinese Anti-cancer Association. Chinese Expert consensus on elective application of hysterectomy in the treatment of cervical HSIL (2022 edition). Chin J Pract Gynecol Obstet. (2022) 38:1108–10. 10.19538/j.fk2022110111

[7] Expert consensus on photodynamic Therapy for Cervical Squamous Intraepithelial Lesions. Chin J Clin Obstet Gynecol. (2026) 27:186–92. 10.13390/j.issn.1672-1861.2026.02.023

[8] Cai H Liu Y Che Y Lou Y Sun H Ma T. Efficacy of photodynamic therapy for cervical intraepithelial neoplasia grade 3: a retrospective study. Photodiagn Photodyn Ther. (2025) 55:104773. 10.1016/j.pdpdt.2025.104773 40849066

[9] Han Q Wang T Wu Z Shi J Zhang X Guo H. Efficacy and safety of photodynamic therapy mediated by 5-ALA for the treatment of CIN3. Photodiagn Photodyn Ther. (2025) 53:104573. 10.1016/j.pdpdt.2025.104573 40194613

[10] Wang X Chen Y Huang J He Q Zhou J. Comparative efficacy of photodynamic therapy and cold knife conization for cervical HSIL. Curr Oncol. (2025) 32:590. 10.3390/curroncol32110590 41294652 PMC12651680

[11] Wang L Liu X Zhang J Song M Liu H Xu Y. Comparison of 5-ALA-PDT and LEEP of cervical squamous intraepithelial neoplasia (CIN2) with high-risk HPV infection in childbearing age women: a non-randomized controlled polit [sic] study. Photodiagn Photodyn Ther. (2024) 46:104044. 10.1016/j.pdpdt.2024.104044 38467338

[12] Li Y Chen J Hu Y Xu Q Jiang R Teng Y. Effects of 5-aminolevulinic acid photodynamic therapy for cervical low-grade squamous intraepithelial lesions with HR-HPV infections. Front Med. (2024) 10:1301440. 10.3389/fmed.2023.1301440 PMC10885802 38404461

[13] Liu Y Medlege H-B Kang Y Wu L Yang W Zhang Y. Clinical efficacy of a new therapeutic option for lower genital tract lesions: 5-ALA photodynamic therapy. Lasers Med Sci. (2024) 39:172. 10.1007/s10103-024-04129-5 38965092

[14] Li X Wei X Liu X Wang N Xu F Liu X. The analysis of HPV integration sites based on nanopore sequencing and the profiling changes along the course of photodynamic therapy. BMC Cancer. (2023) 23:1052. 10.1186/s12885-023-11538-2 37914994 PMC10621124

[15] Xie J Wang S Li Z Ao C Wang J Wang L. 5-aminolevulinic Acid photodynamic therapy reduces HPV viral load via autophagy and apoptosis by modulating ras/raf/MEK/ERK and PI3 K/AKT pathways in HeLa cells. J Photochem Photobiol B. (2019) 194:46–55. 10.1016/j.jphotobiol.2019.03.012 30925276

[16] Hu Z Liu L Zhang W Liu H Li J Jiang L. Dynamics of HPV viral loads reflect the treatment effect of photodynamic therapy in genital warts. Photodiagn Photodyn Ther. (2018) 21:86–90. 10.1016/j.pdpdt.2017.11.005 29155073

[17] Hu ZL Zheng HP Zeng K. Predictors of human papillomavirus persistence or clearance after 5-ALA-based photodynamic therapy in patients with genital warts. Photodiagn Photodyn Ther. (2021) 35:102431. 10.1016/j.pdpdt.2021.102431 34233223

[18] Jin Y Guan Z Wang X Wang Z Zeng R Xu L. ALA-PDT promotes HPV-positive cervical cancer cells apoptosis and DCs maturation via miR-34a regulated HMGB1 exosomes secretion. Photodiagn Photodyn Ther. (2018) 24:27–35. 10.1016/j.pdpdt.2018.08.006 30118903

[19] Wang J Wang Q Chen P Li Q Li Z Xu M. Podophyllotoxin-combined 5-aminolevulinic acid photodynamic therapy significantly promotes HR-HPV-infected cell death. Photodermatol Photoimmunol Photomed. (2022) 38:343–53. 10.1111/phpp.12754 34779024

[20] Taro I Onuma T Kurokawa T Chino Y Shinagawa A YYoshida Y. Evaluating opt-in vaginal HPV self-sampling: participation rates and detection of high-grade lesions (CIN2+) among unscreened Japanese women aged 30–39. Healthcare. (2024) 12:599. 10.3390/healthcare12050599 38470710 PMC10931049

[21] Sami J Lemoupa Makajio S Jeannot E Kenfack B Viñals R Vassilakos P. Smartphone-based visual inspection with acetic acid: an innovative tool to improve cervical cancer screening in low-resource setting. Healthcare. (2022) 10:391. 10.3390/healthcare10020391 35207002 PMC8871553

[22] Ussai S Seyidov T Catalina BN Khomasuridze T. Cervical screening systems in Eastern Europe and Central Asia: a comparative policy evaluation. Healthcare. (2025) 13:2889. 10.3390/healthcare13222889 41302277 PMC12652621

[23] Chen Y Liu L Chen Y Wang Y Yan YF. Prognostic factors influencing the cure rate of 5-aminolevulinic acid photodynamic therapy (ALA-PDT) of high-grade squamous intraepithelial lesions (HSIL): a retrospective cohort study. Photodiagn Photodyn Ther. (2026) 59:105463. 10.1016/j.pdpdt.2026.105463 41941918

[24] Wei Y Niu J Gu L Hong Z Bao Z Qiu L. Effect of clinicopathological characteristics on the outcomes of topical 5-aminolevulinic acid photodynamic therapy in patients with cervical high-grade squamous intraepithelial lesions (HSIL/CIN2): a retrospective cohort study. Biomed. (2024) 12:2255. 10.3390/biomedicines12102255 PMC11504644 39457568

[25] Kim M Choi MC Lee C Na YJ Kim SJ. Long-term outcomes of photodynamic therapy for a positive resection margin after conization for CIN3. Photodiagn Photodyn Ther. (2022) 37:102639. 10.1016/j.pdpdt.2021.102639 34823035

[26] Bao X Chen Y Zhang J Zhang X. Case report: multiphenotypic cervical cancer resembling human papillomavirus-related multiphenotypic sinonasal carcinoma. Front Med. (2024) 11:1507736. 10.3389/fmed.2024.1507736 PMC11655235 39703519

[27] Chen Y Xu Y Zhang ZR Xiong ZH Wu D. 5-aminolevulinic acid-mediated photodynamic therapy effectively ameliorates HPV-infected cervical intraepithelial neoplasia. Am J Transl Res. (2022) 14:2443–51. PMID: 35559391, PMCID: PMC9091109 35559391 PMC9091109

[28] CSCCP. Guidelines for cervical cancer screening in China (part II). Chin J Clin Obstet Gynecol. (2025) 26:88–96. 10.13390/j.issn.1672-1861.2025.01.030

[29] CSCCP. Chinese Expert consensus on the management of CIN2 (2026 edition). Chin J Clin Obstet Gynecol. (2026) 27:89–96. 10.13390/j.issn.1672-1861.2026.01.030

[30] Dobrovolskaya D Asaturova A Badlaeva A Tregubova A Mogirevskaya O Dzharullaeva Z. Role of L1 HPV protein expression in the cytological diagnosis of precancerous cervical lesions. J Clin Med. (2025) 14:3376. 10.3390/jcm14103376 40429372 PMC12112429

[31] Maldonado Alvarado E Osorio Peralta MO Moreno Vázquez A Martínez Guzmán LA Melo Petrone ME Enriquez Mar ZI. Effectiveness of photodynamic therapy in elimination of HPV-16 and HPV-18 associated with CIN I. Photochem Photobiol. (2017) 93:1269–75. 10.1111/php.12769 28380684

[32] Ran R Wang M Li XL Liu Q. A prospective study of photodynamic therapy for cervical squamous intraepithelial lesion. Photodiagn Photodyn Ther. (2021) 34:102185. 10.1016/j.pdpdt.2021.102185 33454394

[33] Zhang T Zhang Y Tang Y Qin L Shen Y Wang B. The effect of high-risk HPV E6/E7 mRNA on the efficacy of topical photodynamic therapy with 5-aminolevulinic acid for cervical high-grade squamous intraepithelial lesions. Photodiagnosis Photodyn Ther. (2022) 39:102974. 10.1016/j.pdpdt.2022.102974 35724936

[34] Waly YM Sharafeldin AB Al-Majmuei A Alatoom M Fredericks S Aloia A-A. Assessment of HPV screening modalities within primary care: a systematic review. Front Med. (2025) 12:1567509. 10.3389/fmed.2025.1567509 PMC12014435 40270492

[35] He RJ Guo WL Hu Y Wu AY Gu L Hong LY. 5-Aminolevulinic Acid photodynamic therapy for non-lesional persistent high-risk HPV infection: a comparative cohort study. Int J Cancer. (2026). 10.1002/ijc.70625 PMC13547979 42332975

[36] Yang WD Jiang GQ Liu YD Zhang LJ Li YJ. Combination of photodynamic therapy and estrogen cream for menopause women with cervical LSIL with HPV infection. Photodiagn Photodyn Ther. (2025) 55:104791. 10.1016/j.pdpdt.2025.104791 40912443

[37] Anton ET-T Matasariu DR Ursache A Anton G-I Balan RA Ursu R-G. Integrating adjuvant HPV vaccination into cervical dysplasia management after LLETZ/conization. J Clin Med. (2026) 15:3424. 10.3390/jcm15093424 42123161 PMC13164224

[38] Han Q Wu ZX Guo HY. Efficacy of 5-aminolevulinic acid photodynamic therapy for the treatment of female lower reproductive tract intraepithelial lesions and its predictive biomarker: DNA methylation. Front Med. (2026) 13:1828763. 10.3389/fmed.2026.1828763 PMC13346238 42428263

[39] Pisarska J Baldy-Chudzik K. MicroRNA-based fingerprinting of cervical lesions and cancer. J Clin Med. (2020) 9:3668. 10.3390/jcm9113668 33203149 PMC7698009

